# Lingual and Jaw Kinematic Abnormalities Precede Speech and Swallowing Impairments in ALS

**DOI:** 10.1007/s00455-018-9909-4

**Published:** 2018-05-17

**Authors:** Bridget J. Perry, Rosemary Martino, Yana Yunusova, Emily K. Plowman, Jordan R. Green

**Affiliations:** 1 MGH Institute of Health Professions, 79/96 13th Street, Charlestown, MA 02109 USA; 2 Department of Speech-Language Pathology, University of Toronto, Toronto, ON Canada; 3 Rehabilitation Sciences Institute, University of Toronto, Toronto, ON Canada; 4 Department of Otolaryngology-Head and Neck Surgery, University of Toronto, Toronto, ON Canada; 5 Krembil Research Institute, University Health Network, 160-500 University Ave, Toronto, ON M5G 1V7 Canada; 6 University Health Network - Toronto Rehabilitation Institute, Toronto, Canada; 7 Biological Sciences, Sunnybrook Research Institute, 160-500 University Ave, Toronto, ON M5G1V7 Canada; 8 Swallowing Systems Core, University of Florida, Gainesville, FL USA; 9 Department of Speech-Language Hearing Sciences and Neurology, University of Florida, Gainesville, FL 32610 USA; 10 Program in Speech and Hearing Bioscience and Technology, Harvard University, 260 Longwood Avenue, Boston, MA 02115 USA

**Keywords:** Deglutition, Deglutition disorders, Amyotrophic lateral sclerosis, Kinematics, Electromagnetic articulography

## Abstract

Early identification of bulbar involvement in persons with ALS is critical for improving diagnosis and prognosis; however, efficacious diagnostic markers have not yet been identified. The purpose of this study was to determine whether kinematic changes of the tongue and jaw during swallowing, measured using 3D electromagnetic articulography (EMA), predate clinically identifiable symptoms of speech and swallowing impairment in persons diagnosed with ALS. Data were collected from 16 adults diagnosed with ALS and 18 neurotypical controls. Groups were aged matched. Eligible participants with ALS were tolerating an unrestricted diet (FOIS = 7), produced intelligible speech (> 97%), and had a speaking rate greater than 150 words per minute. Participants completed a 3-mL water swallow task, during which EMA recorded kinematic measures of the anterior and posterior regions of tongue including lingual speed, range of motion, duration, coordination, and efficiency. Jaw speed and range of motion were also recorded. Persons diagnosed with ALS demonstrated reduced posterior lingual range of motion (11.40 mm ± 4.01 vs. 16.07 mm ± 5.27), slower posterior lingual speeds (83.67 mm/s ± 47.96 vs. 141.35 mm/s ± 66.54), increased lingual movement duration (13.46 s ± 6.75 vs. 9.21 s ± 3.28), and reduced lingual coordination (0.04 s ± 0.11 vs. 17 s ± 0.19) during the 3-oz water swallow task compared to controls. Persons diagnosed with ALS demonstrated increased range of motion (9.86 mm ± 5.38 vs. 6 mm ± 3.78) and increased jaw speed (68.62 mm/s ± 50.13 vs. 34.72 mm/s ± 17.75) during swallowing compared to controls. The current findings suggest that changes in lingual and jaw motor performance during a simple water swallow task are present in persons with ALS who are pre-symptomatic of clinically detectable bulbar impairment.

## Introduction

Amyotrophic lateral sclerosis (ALS) is a fatal neuromuscular disease characterized by the rapid degeneration of both upper and lower motor neurons of the brain and spinal cord, resulting in progressive deterioration of muscle function throughout the body [1]. The progressive loss of motor function over bulbar structures, such as the face, mouth, pharynx, and larynx, results in speech and swallowing impairments in most individuals with ALS [1]. Among the bulbar muscles, the muscles of the tongue appear to be disproportionally affected by ALS [2, 3] and, at the time of ALS diagnosis, tongue weakness is a prognostic indicator of survival in ALS [4]. Although the link between tongue weakness and survival is likely due to swallowing impairments, only a few studies have characterized the tongue dysfunction during speech and swallowing in ALS [5, 6]. Such knowledge may be essential for understanding the mechanisms of dysphagia in ALS and for the early identification of patients at risk for aspiration. Per Luchesi et al., delaying the implementation of swallowing management can be a risk factor for malnutrition, which has been found to negatively impact survival in this population [1].

The extant literature on swallowing impairments due to ALS is based on videofluoroscopic observations of bolus transport through the oral cavity, pharynx, and upper esophageal sphincter [7–9]. Oral swallowing deficits have been observed early in the disease process and characterized as difficulties with mastication, oral preparation, and lingual transport [7–10]. Lingual transport of the bolus during normal swallowing has been described using a variety of motion capture techniques including videofluoroscopy [11], ultrasound [12], slow-motion cinematography [13], and more granular point-tracking techniques such as X-ray microbeam [14, 15] and electromagnetic articulography (EMA) [16].

Point-tracking techniques have been used to quantify the spatiotemporal coordination of lingual transport in neurotypicals. Using X-ray microbeam, Wilson and Green [15] reported that lingual transit time, the temporal difference between the onset of anterior tongue movement and the onset of posterior tongue movement, in healthy control participants was 168 ms during a 10-cc discrete water swallow. Steele and Van Lieshout [16] explored lingual movements during swallowing in eight healthy adults using EMA, and concluded that EMA was able to adequately capture movements of the oral tongue blade, body, and dorsum during the swallow and was thereby an effective tool for tracking tongue movements during deglutition. Steele [16] and Wilson and Green [15] both suggested that point-tracking methods of quantifying tongue movements, such as EMA and X-ray microbeam, would be useful for tracking changes in tongue motor performance in populations with lingual impairments.

The purpose of this study was to determine if changes in tongue and jaw movement during liquid swallows were present in persons with ALS who were pre-symptomatic of clinically detectable bulbar impairment. We hypothesized that during liquid intake these patients will present with changes in tongue and jaw kinematics relative to neurotypical controls.

## Methods

### Participants

Data were collected as a part of a larger ongoing study investigating longitudinal speech changes in persons with ALS. Sixteen participants with ALS and 18 neurotypical controls were selected based on a priori inclusion criteria. Participants in the patient group had to be diagnosed with ALS by a neurologist following the criteria defined by the El Escorial Criteria from the World Federation of Neurology [17] with no history of any other neurological impairment. Participants in the neurotypical control group had to have no history of any known neurological, cognitive, speech, or swallowing impairment. Participants in the ALS group needed to be clinically asymptomatic of speech and swallowing impairments, defined with a self-report of 7 on the Functional Oral Intake Scale [18], indicating they were tolerating an unrestricted diet. Additionally, participants were required to pass the 3-oz water swallow test [19]. Speech intelligibility needed to be within normal limits (> 97%) as determined by a blinded research assistant using the Speech Intelligibility Test and speaking rate needed to be > 150 words per minute [20]. For both groups, tongue and jaw movement data needed to be free of movement-tracking artifact, including missing movement traces and movement mistrackings, during the swallowing task. Groups were statistically matched for age. Sex was not controlled for, because a study by Steele and Van Lieshout found no significant differences in lingual movements between males and females [21].

### Task

For the swallowing task, participants were seated upright in a supportive chair and provided with 3 oz of water in a cup in the manner they typically drink (cup sip or use of straw). They were instructed to drink all of the water in consecutive sips without stopping either using a cup or straw. Instructions were provided both verbally and in written form.

### Instrumentation

**Fig. 1:**
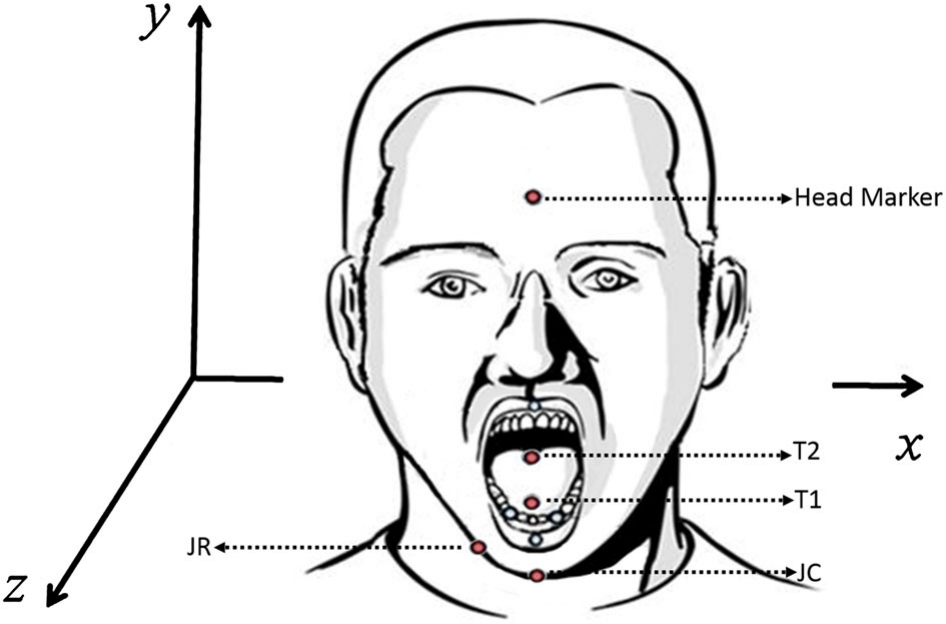
EMA sensor placement and orientation. Red sensors indicate sensors used for data analysis

An electromagnetic tracking device (Wave; Northern Digital, Inc.) was used to record tongue and jaw movements during the swallow. The system used a combination of 5 and 6-degree-of-freedom (5DOF and 6DOF) sensors to record labial, lingual, and jaw motions in a calibrated volume (30 × 30 × 30 cm). A 6DOF sensor was securely placed on the head to serve as a reference sensor to register head movements and re-express the 3D tongue and jaw data relative to a head-based coordinate system using the Northern Digital, Inc. system default settings. Prior research in healthy populations has shown that swallow function is not affected by tongue sensor placement [22] and that the use of only two sensors is adequate to determine differences in movements between anterior and posterior portions of the tongue [15]. Two 5DOF tongue sensors were attached to the tongue (see Fig. 1): one at midline, approximately 1 cm posterior to the tongue tip (T1), and the second approximately 4 cm posterior to the tongue tip (T2) using PeriAcryl Oral Tissues Adhesive (GluStitch Inc.), a non-toxic dental glue. At the time of this study, EMA technology was still in development; therefore, a combination of comparable sensor configurations was used on the jaw, mainly one 6DOF or two 5DOF sensors. Following data collection, within group *t* tests between sensor types were completed to check for sensor differences. Because sensor effects were not observed in either jaw speed or range of motion, data from each jaw sensor type (6DOF and 5DOF) were pooled for statistical analysis.

### Data Processing

A Matlab-based program, SMASH [23], was used to post-process and analyze the tongue and jaw movement time series. All data were manually checked for missing data and movement artifacts, and a low-pass filter at 10 Hz was applied to remove high-frequency noise from the signals. The first and last lingual cycles from each participant were excluded to avoid extraneous tongue and jaw movements associated with the initiation and completion of the swallow task.

### Biomechanical Measures

Kinematic measures of tongue and jaw movement were extracted from movements along the vertical (*y*) axis of the frontal plane (see Fig. 1):

1. Range of movement was calculated as the difference between the maximum and minimum values of the vertical distance trace for the tongue and jaw separately [24].
2. Maximum speeds of movement of tongue and jaw were calculated as the maximum values of the first derivatives of, respectively, the vertical tongue and jaw movement distances [24].
3. Movement duration of the tongue was calculated as the time between the movement onset and offset for each of the anterior and posterior tongue sensors [24].
4. Tongue coordination was calculated using a cross-correlation analysis (Fig. 2), and was defined as the temporal lag between the initiation of anterior lingual elevation and the initiation of posterior lingual elevation [15, 25].

**Fig. 2:**
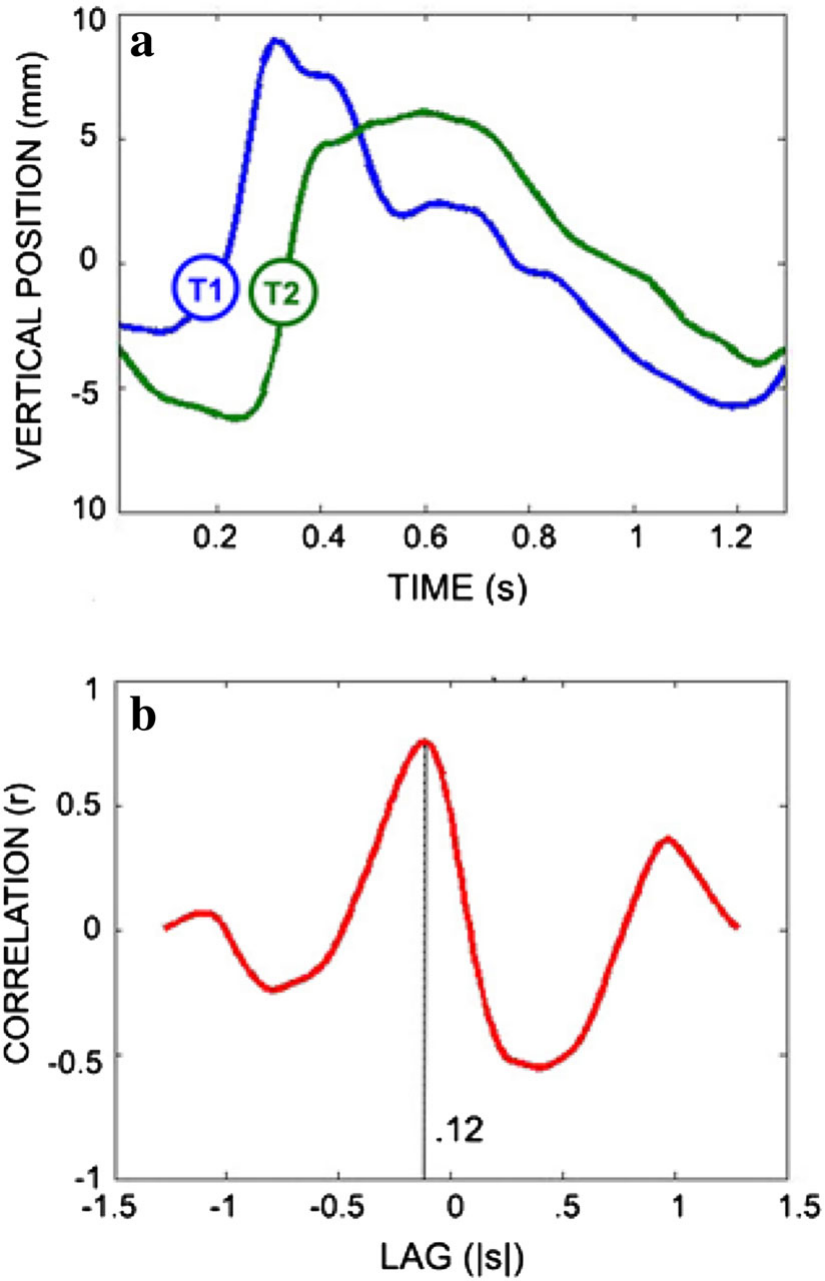
**a** Times series of anterior (T1) and posterior (T2) lingual movement during swallowing, **b** interval between the motions of T1 and T2 (lag)
5. Lingual efficiency was calculated as the number of lingual movement cycles completed during the 3-oz water swallow test.

### Statistical Analysis

Given the small sample size and non-normal distribution of the data, non-parametric Mann–Whitney *U* tests were used to address research questions. Statistical analyses were run using the *R* statistical software (RStudio Team 2015). A Holm–Bonferroni correction was applied to reduce the familywise error rate for multiple comparisons [26]. This correction uses a sequential method for rejecting null hypotheses. Following Mann–Whitney *U* tests, *p* values were ranked from smallest to largest and compared to significance levels *α*/*n*, *α*/(*n* − 1), … *α*/1, where *α* was the target alpha level and *n* was the total number of tests performed. Using this method for the three planned contrasts of the tongue, the test with the lowest *p* value was compared to a significant level of *α* = 0.05/3 = 0.016, the test with the second lowest *p* value was compared to a significance level of *α* = 0.05/(3 − 1) = 0.025, and the final *p* value was compared to significance levels of *α* = 0.05. For the two planned contrasts of the jaw, the test with the lowest *p* value was compared to a significant level of *α* = 0.05/2 = 0.025 and the final test was compared to a significance level of *α* = 0.05. Between-group differences in lingual coordination were determined using a single Mann–Whitney *U* test on lag times derived from a cross-correlation analysis. This method provides robust protection against Type I errors while maintaining higher power than a classic Bonferroni correction [26].

## Results

### Lingual Movement

**Fig. 3:**
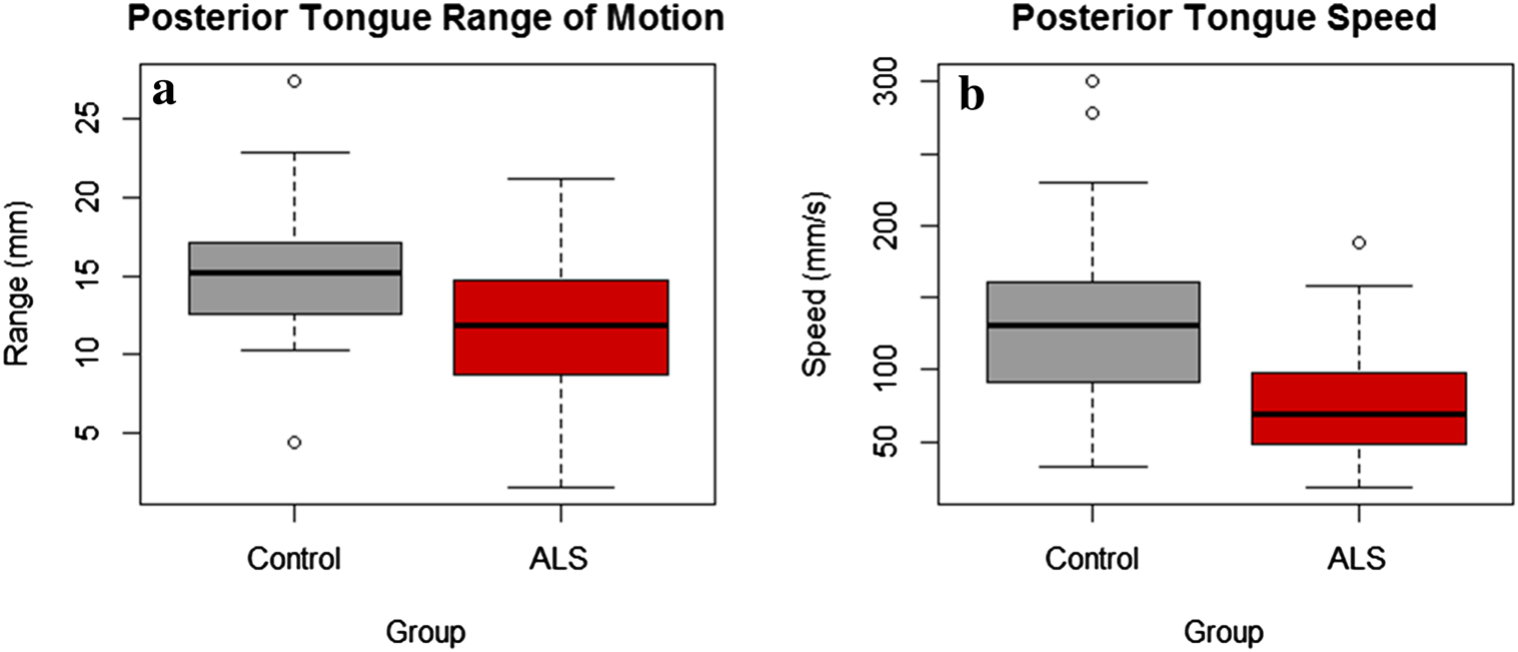
**a** Between-group differences in posterior tongue range of motion, **b** between-group differences in posterior tongue speed

There were no statistically significant group differences in anterior lingual range of motion or in anterior lingual speed. Posterior lingual range of motion was significantly reduced (11.40 mm ± 4.01 vs. 16.07 mm ± 5.27, *p* = 0.021) in persons with ALS than in neurotypical controls (Fig. 3a). Posterior lingual speed was significantly slower (83.67 mm/s ± 47.96 vs. 141.35 mm/s ± 66.54, *p* = 0.008) in persons with ALS than in neurotypical controls (Fig. 3b).

**Fig. 4:**
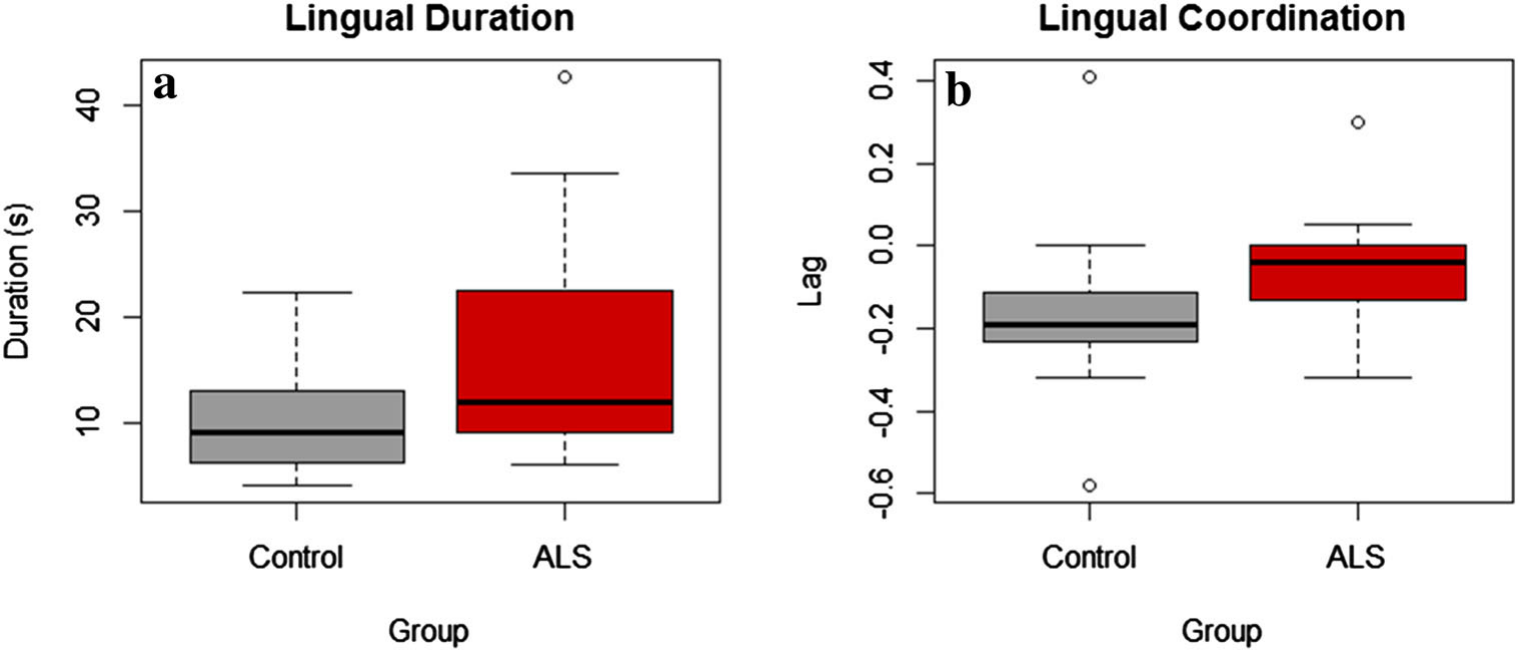
**a** Between-group differences in lingual duration, **b** between-group differences in lingual coordination

Lingual coordination, as indexed by the temporal lag measure, was significantly shorter (0.04 s ± 0.11 vs. 0.17 s ± 0.19, *p* = 0.005) in persons with ALS than in the neurotypical controls (Fig. 4a). The duration of lingual movement over the course of the task was significantly longer (13.46 s ± 6.75 vs. 9.21 s ± 3.28, *p* = 0.046) in persons with ALS than in neurotypical controls (Fig. 4b). No significant group differences were observed in lingual efficiency.

### Jaw Movement

**Fig. 5:**
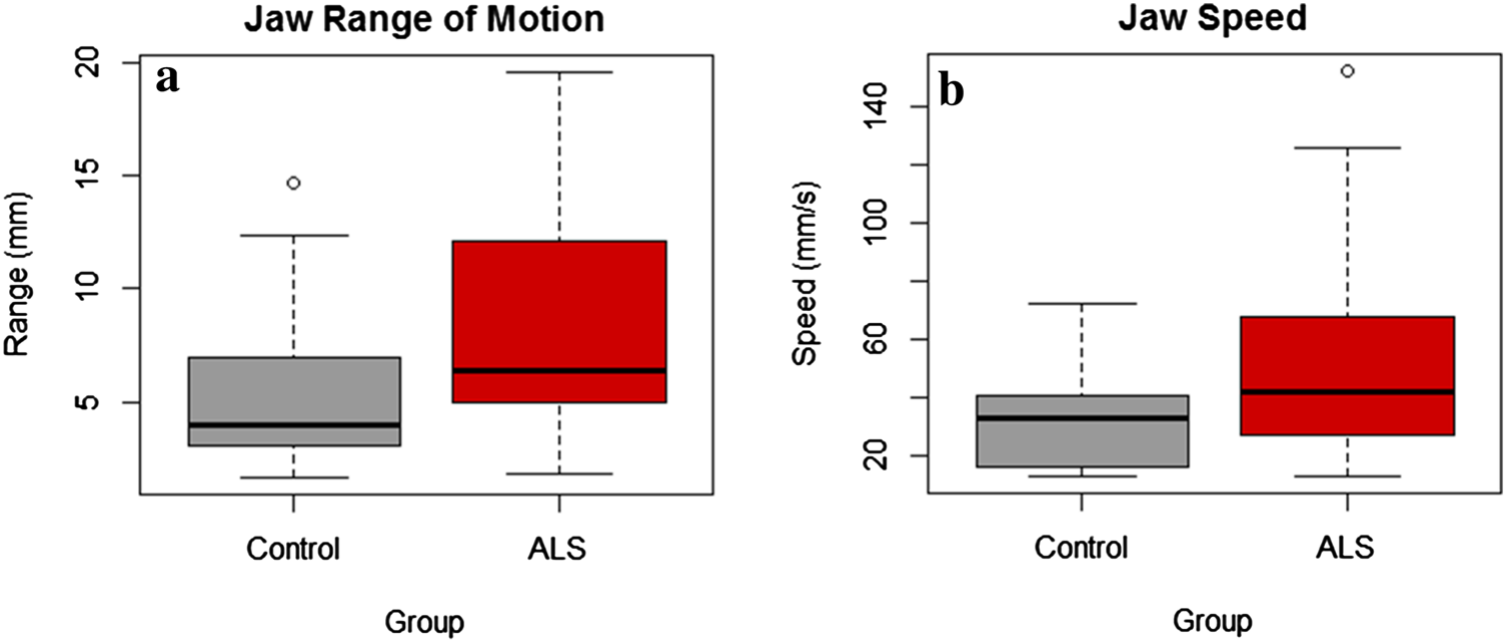
**a** Between-group differences in jaw range of motion, **b** between-group differences in jaw speed

Results of group comparisons on jaw range of motion and jaw speed revealed that jaw range of motion was significantly greater (9.86 mm ± 5.38 vs. 6 mm ± 3.78, *p* = 0.043) and jaw movements were significantly faster (68.62 mm/s ± 50.13 vs. 34.72 mm/s ± 17.75, *p* = 0.021) in persons with ALS than in neurotypical controls (Fig. 5a, b).

## Discussion

The results of this study suggest that kinematic differences between ALS and neurotypical patients in the tongue and the jaw during swallowing are detectible prior to clinically discernable speech and swallowing impairments. This finding has several clinical implications including improving early detection of swallowing impairment in persons with ALS.

### Early Kinematic Indicators of Tongue and Jaw Involvement: Speed and Range of Motion

Using 3D EMA to measure tongue speed, tongue range of motion, and tongue coordination, we detected multiple changes in lingual motor function (i.e., decreases in posterior tongue speed and posterior tongue range of motion) prior to the onset of speech or swallowing impairments. The suggestion that lingual coordination is affected during the early stages of ALS is supported by our observations that in comparison to the neurotypical participants, the participants with ALS demonstrate longer swallowing durations but shorter lags between the movements of the anterior and posterior tongue. Shorter lags are interpreted as increased dependence between the movement of different tongue regions and thus suggestive of constrained lingual coordination.

These findings corroborate prior findings that suggest changes in tongue function during the swallow are present in persons with ALS without clinically observable bulbar impairment. Specifically, two prior studies have used videofluoroscopy to describe changes in swallow function in persons with ALS early in the disease process. A study by Murono et al. [8] described impairments in bolus transport/lingual motion, oral residue, pharyngeal contraction, and pharyngeal residue in five persons presenting without bulbar impairment at the time of diagnosis. Higo et al. [27] reported videofluoroscopic findings including delayed bolus transit and pharyngeal residue in persons with ALS but no bulbar symptoms. Future work is, therefore, needed to determine the added value of kinematic analysis alongside VFSS-based clinical assessments of swallowing function. We speculate that for persons with neurodegenerative diseases, biomechanical analyses, like the one used in this study, will be more sensitive and responsive to impairments in lingual movements than are clinically based measures of tongue function.

### Changes in the Posterior Tongue May Precede Changes in the Anterior Tongue

Our study found early motor changes in posterior tongue but not in anterior tongue during sequential liquid swallowing, a finding that suggests that posterior tongue is affected earlier in the disease process than is anterior tongue. A similar finding was reported in a study investigating tongue movements during speech in persons with ALS [5]. In that study, the spatiotemporal coupling of mid-posterior tongue regions was found to be impaired, while anterior tongue regions remained largely unaffected in persons with moderate speech impairments [5]. Interestingly, these findings differ from anatomical studies of the tongue in persons with ALS, which have reported a disproportionate degree of degeneration of muscle fiber groups [28], and increased atrophy, fat, and fibrosis [29, 30] in the anterior tongue relative to posterior tongue. During swallowing, movement of the anterior tongue is restricted when compared to movements of the posterior tongue, as the anterior tongue forms a seal against the palate, while the posterior tongue propels the bolus into the pharynx [11–13]. As a result, movements of the posterior tongue evoke larger displacements and greater speeds and, therefore, may be more likely to reveal motor deficits [16, 21]. In contrast, movement deficits in anterior tongue may be masked during swallowing because the anterior tongue is fixed against the palate.

### Jaw Movements May Begin to Compensate for Lingual Dysfunction Early in the Disease Process

Another robust, but perhaps unexpected finding was the increase in jaw speed and range of motion in persons with ALS, which coincided with decreases in tongue range of motion. This finding is similar to that reported in speech [6, 31, 32]. The authors in these studies speculated about a potential compensatory role for the jaw in response to declining tongue function for speech. Our study extends these findings to swallowing, suggesting that even in the early stages of the disease jaw movements begin to compensate for tongue dysfunction.

### Clinical Implications

Understanding the limitations of current best practice is an important first step toward improving early diagnosis of bulbar impairment. At present, clinical assessment of bulbar involvement relies heavily on subjective clinician observations, more objective clinician ratings, and patient reporting. A recent study by Allison et al. [33] found that instrument-based measures of speech were more sensitive to early speech changes in persons with ALS than were measures based on patient self-report and clinician ratings. Our study adds to this work by concluding that in the early stages of the disease process, objective analyses of tongue movement may be useful for identifying pre-symptomatic bulbar motor changes.

The early detection of slowed lingual transport on a 3-oz water swallow task motivates additional research into the use of timed tasks in clinical settings. Prior work by Langmore and Lehman [34] and Rong et al. [35] suggests that rate-based tasks, such as alternating motion rate tasks (AMRs), are sensitive to disease-related changes early in the disease process. Additionally, prior work has shown that simple stop-watch-based measures of the chewing sequence duration can be reliably and accurately estimated by trained clinicians [36]. In conjunction with results from our study, these findings suggest that the timing of oral behaviors such as swallowing, chewing, and speech may be a low-tech and reliable method for benchmarking bulbar motor involvement.

The instrumentation-based techniques used in this study may also be useful for evaluating the effectiveness of therapeutic interventions early in the disease process and for stratifying patients, which is needed to make decisions about the appropriateness of, for example, exercise-based interventions. Plowman et al. [37], for example, have suggested that mild- to moderate-intensity exercise may be beneficial if carefully applied *early* in the disease process to maintain the vital functions of breathing and airway protection in ALS.

At present, EMA or other 3D point-tracking methodologies of the tongue are not clinically feasible because the equipment is relatively expensive and the procedures for affixing tongue are time consuming. This technology, however, is well suited to serve as a “gold standard” for future efforts directed toward validating clinician-administered assessment tools of speech and swallowing function.

## Limitations and Future Work

Natural history studies are needed to improve our understanding of the impact of lingual impairment on swallow physiology and to improve prognostic capabilities of quantitative swallowing assessments. Future studies aim to explore EMA’s responsiveness to changes in lingual movements in early stages of the disease process as well as over the course of the disease. One limitation to this study is that only one swallowing task was administered—a single swallowing trial may not be reflective of overall performance and is probably inadequate for assessing the impact of fatigue, which can play a role in swallow performance over the course of a meal. Future studies should expand upon a single 3-oz sequential water swallow task to include varying liquid and solid consistencies and amounts over longer trial periods. We expect this work will help to guide physiologically based therapeutic exercises aimed at prolonging swallowing function in this population. Additionally, kinematic assessment of bulbar function during the swallow may help identify critical markers of bulbar decline, which will improve the management of risks associated with dysphagia [38, 39] and improve timing of the implementation of diet modifications and non-oral feedings [38, 39].
